# Genomic characterization of Volzhskoe tick virus (*Bunyaviricetes*) from a *Hyalomma marginatum* tick, Hungary

**DOI:** 10.1038/s41598-024-69776-8

**Published:** 2024-08-15

**Authors:** Gábor Földvári, Zsófia Tauber, Gábor Endre Tóth, Dániel Cadar, Alexandra Bialonski, Balázs Horváth, Éva Szabó, Zsófia Lanszki, Brigitta Zana, Zsaklin Varga, Fanni Földes, Gábor Kemenesi

**Affiliations:** 1 Institute of Evolution, HUN-REN Centre for Ecological Research, Konkoly-Thege Miklós út 29-33, Budapest, 1121 Hungary; 2 Centre for Eco-Epidemiology, National Laboratory for Health Security, Konkoly-Thege Miklós út 29-33, Budapest, 1121 Hungary; 3 Doctoral School of Biology, Institute of Biology, Eötvös Loránd University, Budapest, Hungary; 4 Szentágothai Research Centre, National Laboratory of Virology, University of Pécs, Pécs, 7624 Hungary; 5 Institute of Biology, Faculty of Sciences, University of Pécs, Pécs, 7624 Hungary; 6 Bernhard Nocht Institute for Tropical Medicine, Hamburg, Germany; 7 School of Biomedical Sciences, University of Plymouth, Plymouth, PL4 8AA UK

**Keywords:** *Hyalomma marginatum*, Tick, Volzhskoe tick virus, *Bunyaviricetes*, Illumina-based viral metagenomic sequencing, Virology, Metagenomics

## Abstract

*Hyalomma marginatum*, a vector for the high-consequence pathogen, the Crimean–Congo hemorrhagic fever virus (CCHFV), needs particular attention due to its impact on public health. Although it is a known vector for CCHFV, its general virome is largely unexplored. Here, we report findings from a citizen science monitoring program aimed to understand the prevalence and diversity of tick-borne pathogens, particularly focusing on *Hyalomma* ticks in Hungary. In 2021, we identified one adult specimen of *Hyalomma marginatum* and subjected it to Illumina-based viral metagenomic sequencing. Our analysis revealed sequences of the uncharacterized Volzhskoe tick virus, an unclassified member of the class *Bunyaviricetes*. The in silico analysis uncovered key genetic regions, including the glycoprotein and the RNA-dependent RNA polymerase (RdRp) coding regions. Phylogenetic analysis indicated a close relationship between our Volzhskoe tick virus sequences and other unclassified *Bunyaviricetes* species. These related species of unclassified *Bunyaviricetes* were detected in vastly different geolocations. These findings highlight the remarkable diversity of tick specific viruses and emphasize the need for further research to understand the transmissibility, seroreactivity or the potential pathogenicity of Volzhskoe tick virus and related species.

## Introduction

Most of the emerging human viral diseases have zoonotic origins, and approximately 40% of these are vector-dependent^1^. Strong correlations exist between the distribution and emergence of tick-borne viruses (TBVs), the vector's population dynamics, and the expanding host range^2^. Ticks are hematophagous vectors capable of spreading viruses to both human and animal hosts. Empirical evidence indicate that climate change affects the dynamics of emerging infectious diseases and especially vector-borne pathogens, via changing the distribution of pathogen hosts and arthropod vectors^3^.

*Hyalomma marginatum* is a major threat to both human and animal health because it is known to carry *Rickettsia aeschlimannii* and Crimean-Congo hemorrhagic fever virus (CCHFV)^4^. Migratory birds carry larval and nymphal *Hyalomma* ticks, leading to their occurrence in novel places [^5,6^]. There were sporadic reports of immature *Hyalomma* ticks found on a hedgehog and song birds and two adult *Hyalomma rufipes* ticks from cows during the last decade in Hungary^7–10^. Locally acquired immatures of *Hy. rufipes* were recently found on birds in the country raising the possibility of a local population^11^. Additionally, there is evidence that both humans^12^ and animals [^13,14^] have antibodies against the CCHFV virus in Hungary.

As the geographical distribution of their vectors changes, viruses, especially Crimean–Congo Hemorrhagic fever virus of the class *Bunyaviricetes* cause major concerns for human health and livestock^15^.

Segmented, linear, single-stranded negative sense RNA genomes are common genome features in the class *Bunyaviricetes*. Multiple copies of the nucleocapsid protein (NP) encapsulate the genomes and the enveloped virions coated by glycoproteins. In general, bunyaviruses have three genome segments, referred to as large (L), medium (M), and small (S) segments. However, some species in the *Arenaviridae*, *Wupedeviridae*, and unassigned families have bisegmented genomes, whereas viruses with four to six segments are known in other families. It should be noted that RNA-dependent RNA polymerase (RdRp) is also packed in the virion^16^.

Taking advantage of the rapid development of next-generation sequencing (NGS) methods in recent years, we are now able to take a closer look at the virome of a tick. This is particularly important because many novel viral sequences have been identified in ticks from various species; hence, ultimately, we can better detect the spread of vector-borne diseases as well.

It has been possible to find a very large group of insect-specific viruses (ISVs) through metagenomics-based virologic surveillance, in addition to finding TBVs early on. These viruses are only known to infect insects (or arthropods), not mammals^17^, and can engage in negative^18^ or positive^19^ relationships with pathogenic agents.

We launched a citizen-science project in 2021 (https://kullancsfigyelo.hu) to document and monitor the emergence and potential establishment of *Hyalomma *spp.^20^. Participants collected two adult male *Hyalomma *spp. ticks within the first seven months: a *Hy. marginatum* tick from a dog and a *Hy. rufipes* tick from a cow. In an attempt to take preventive measures by applying the DAMA (Document, Assess, Monitor, Act) protocol^21^, we characterized the virome of the *Hy. marginatum* adult specimen by high-throughput metagenomic sequencing.

## Results

### Volzhskoe tick virus genome

The assembled sequence of the M and L segments showed 91.53% and 93.56% identity with the Volzhskoe tick virus from Russia (MN542371, MN542370) on the nucleotide level, respectively. It is common for species of the class *Bunyaviricetes* to have a tripartite genome, but in the case of the Volzhskoe tick virus, the S segment was not present in the sequenced virome or was so different that it could not be identified as a viral sequence. We deposited the resulting sequences in GenBank under accession numbers OQ849228 (M segment) and OQ849227 (L segment).

**Figure 1:**
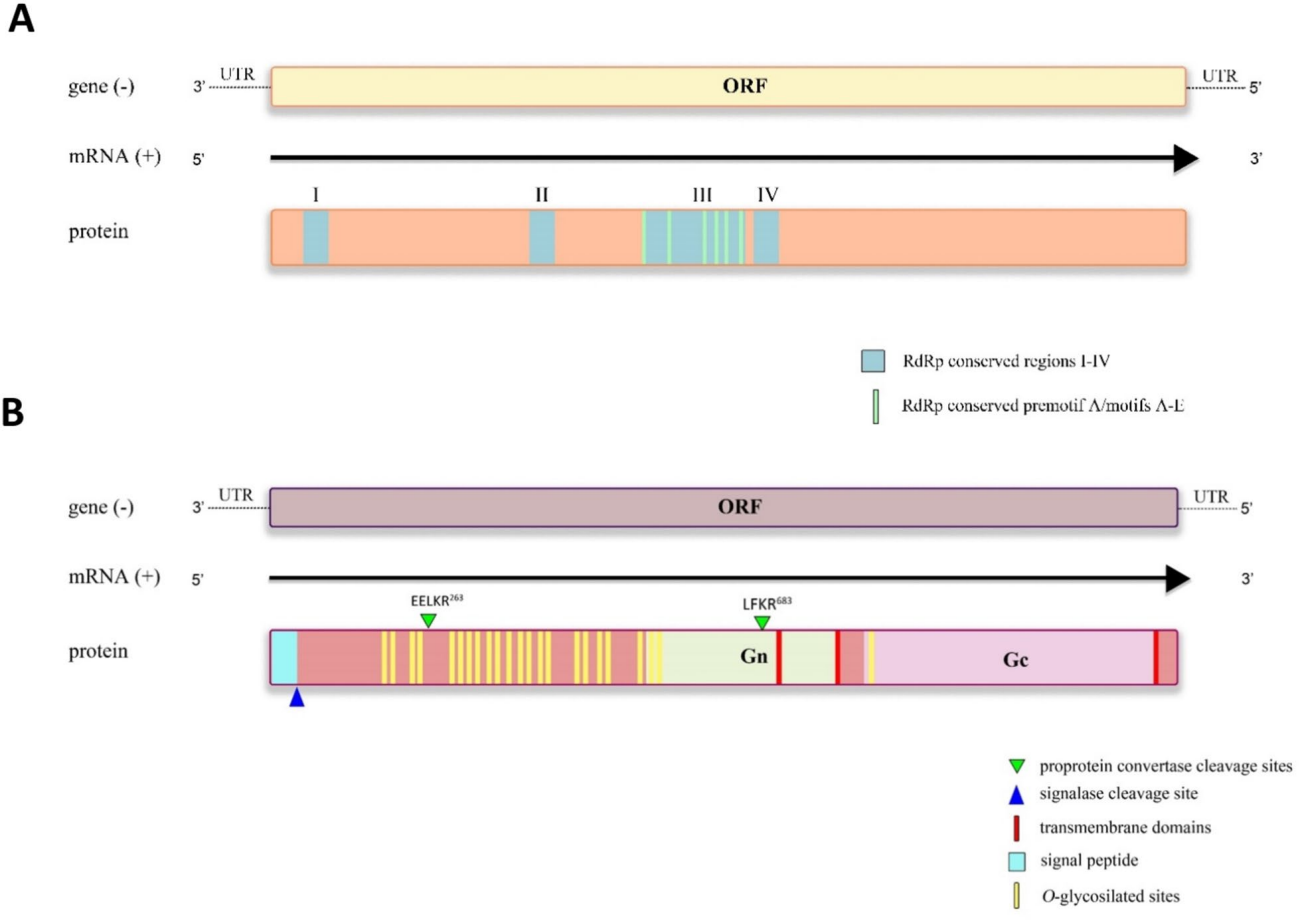
Schematic map of segment L and the encoded RNA-dependent RNA (**A**) polymerase and segment M and the encoded Glycoprotein precursor (**B**).

In the assembled, manually corrected sequence (8804 bases), a single ORF with a predicted amino acid sequence of 2876 aa in length was discovered within the L segment of the Volzhskoe tick virus. Previous studies have identified four conserved regions (I–IV) in the L protein of bunyaviruses^22^. One of these regions, known as the functional core of the polymerase, consists of six conserved motifs (premotif A and motifs A-E)^23^. We were able to find all four conserved regions (Fig. 1a) in the predicted amino acid sequence. The RdRps of selected bunyaviruses were aligned, and the Volzhskoe tick virus was found to contain the expected conserved motifs, including Motif A (DxxKWx), Motif B (QGxxNxxSS), Motif C (SDD), Motif D (KK), and Motif E (EFxSx), with our sequence containing serine (S) instead of glutamic acid (E) in this motif. We also found premotif A, which has three basic residues (K, R, and R/K) and a downstream glutamic acid (E) (Figure S1). In Motif C, the two conserved aspartic acid (D) residues are essential for catalytic activity^24^.

The 4258 base long assembled and corrected M segment of the Volzhskoe tick virus encodes a bunyavirus glycoprotein complex (GPC) with a length of 1373 aa. InterPro and HMMER searches found only the Gc in the predicted polyprotein, from residue 860 to 1372, but using JackHMMER, we were able to predict the position of Gn (residue 589–825) as well. The GPC was predicted to have an N-terminal signal peptide together with a signal peptide cleavage site between the 16 and 17 residues. Three transmembrane helices were detected in the GPC: one between residues 735 and 744, the second between 839 and 854, and the third at 1351–1372. We found rich O-glycosylation sites in the N-terminal part of the predicted GPC. The sites predicted to undergo proteolytic cleavage in the Volzhskoe tick virus GPC are shown in Fig. 1b.

**Figure 2:**
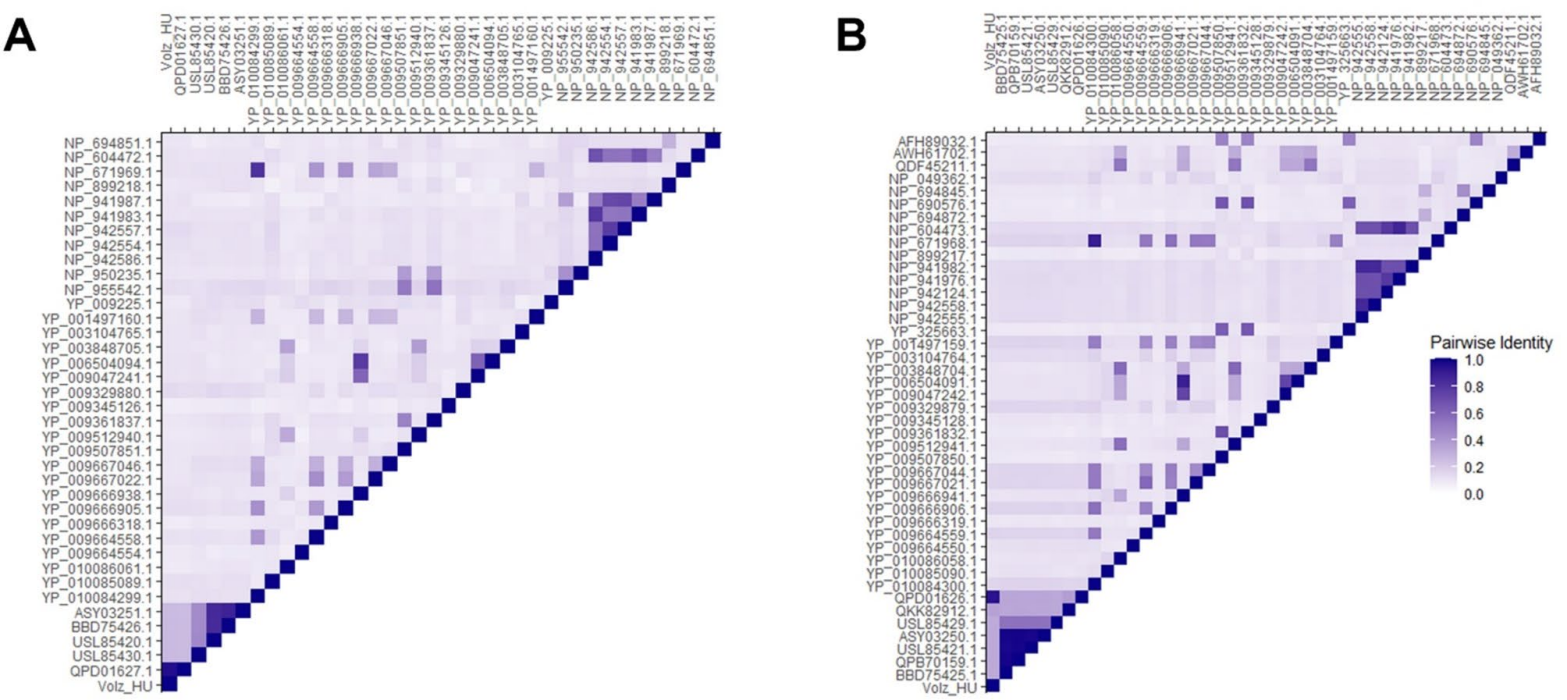
Pairwise amino acid identity matrix of Bunyaviricetes GPCs (**A**) and RdRps (**B**), virus accession numbers are given on Figure S2 and in Tables S1–S2.

**Figure 3:**
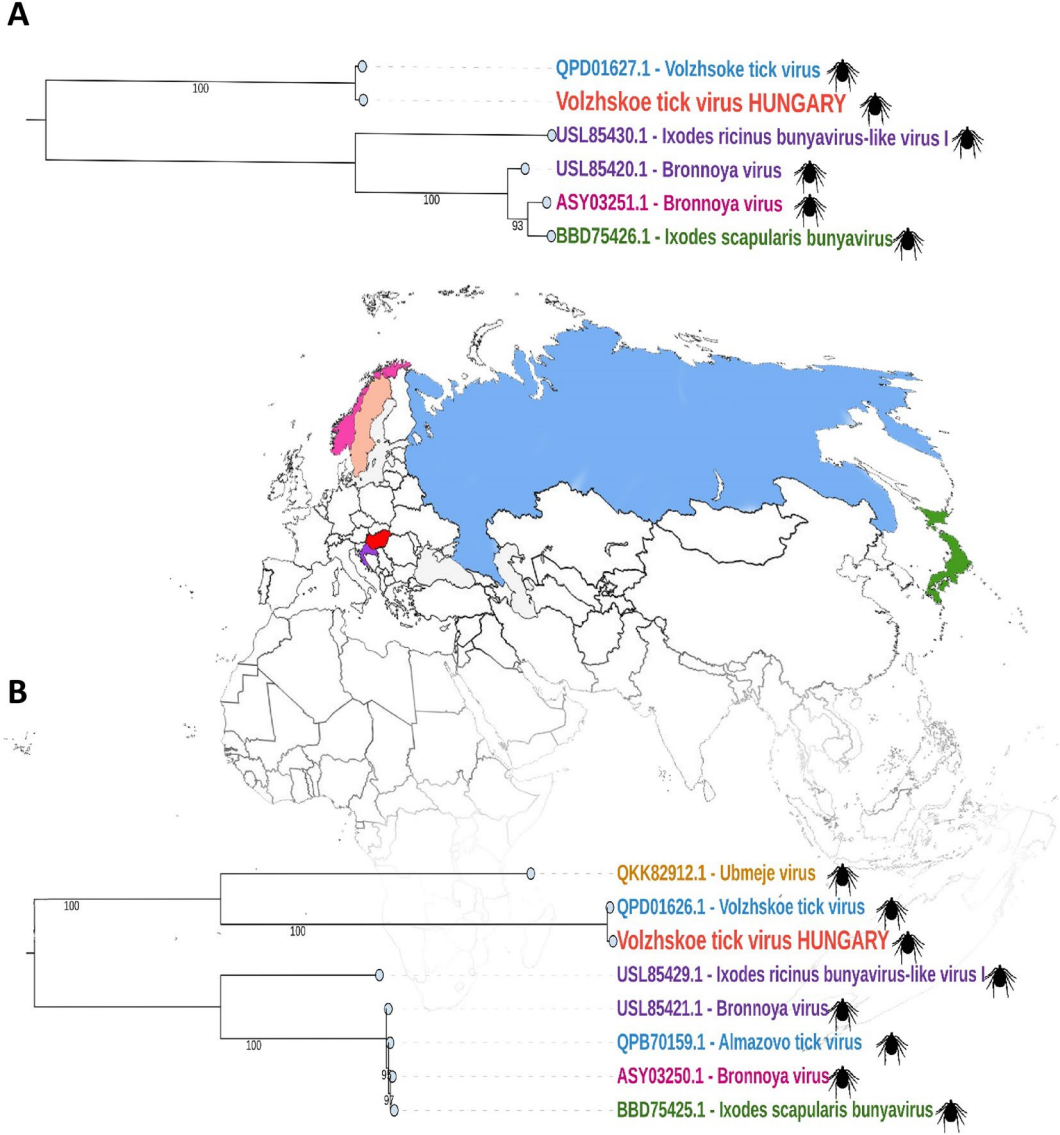
Maximum likelihood phylogenetic trees based on the segment M (**A**) and segment L (**B**) of the closest relatives and the newly identified Volzhskoe tick virus (red). The colours indicate the countries where the strains were discovered: Croatia (purple), Hungary (red), Japan (green), Norway (pink), Russia (blue), Sweden (orange).

According to the phylogenetic analysis, the closest relatives of the Volzhskoe tick virus are other unclassified bunyaviruses. For initial analysis, we selected sequences among the families of the class *Bunyaviricetes*. During the analysis of the M segment and the L segment, 38 and 44 species were aligned, respectively. For each protein, the Volzhskoe tick virus showed an identical pattern of clustering and based on the results, these unclassified species are on the same branch, this branch forms a monophyletic clade with the family *Peribunyaviridae* (Figure S2). We generated pairwise-identity matrices for the M and L segments across the class *Bunyaviricetes* and these confirmed the results that our phylogenetic analyses indicated. Based on the amino acid sequence alignments, the highest pairwise identity between the discovered Volzhskoe tick virus and representative species of *Bunyaviricetes* ranged from ~ 45% (*Ubmeje virus*) to ~ 90% (Volzhskoe tick virus—Russia) (Fig. 2). Our second phylogenetic analysis focused only on the closest relatives of the Volzhskoe tick virus; these trees are colored according to the geographic locations where the species were sequenced (Fig. 3). Of course, both of the newly sequenced segments demonstrated the closest evolutionary relationship to the Volzhskoe tick virus segments sequenced in Russia. Based on the RdRp, these two strains form a strongly supported monophyletic clade with the Ubmeje virus found in Sweden. All of the closely related unclassified bunyaviruses were found in various species of ticks.

## Discussion

Our goal was to extend our knowledge about viral diversity within the non-endemic *Hy. marginatum* tick since we only know a small fraction of viral diversity globally and this poses a threat to anticipate disease emergence^25^. Some viruses have been shown to have the potential to regulate arbovirus transmission in mosquitoes^17^, and we have limited information about tick-specific viruses. The metagenome of our tick sample did not contain CCHFV. The taxonomic classification of the short reads revealed that the most abundant virus was the Volzhskoe tick virus, a member of the *Bunyaviricetes*. The class *Bunyaviricetes* includes several known pathogens, such as CCHFV, La Crosse virus, and Rift Valley fever virus. We could assemble the M and L segments, but we were unable to identify the S segment in the metagenome. Recent metagenomic analyses have shown that Ixodes ricinus bunyavirus-like virus 1 (IRBV1) and the Croatian strain of *Bronnoya virus* also lack the S segment^26^, which, together with our data, suggests that this may be a characteristic feature of unclassified tick-specific bunyaviruses, as these species are the closest relatives of Volzhskoe tick virus based on our phylogenetic trees. However, it is also possible that these segments may be different enough from known ones that they cannot be identified as viral segments.

The predicted amino acid sequence from the ORF of the L segment of the newly identified Volzhskoe tick virus revealed typical *Bunyaviricetes* RdRp regions. In Region III, known as the functional core of the molecule, the necessary six conserved motifs^24^ were displayed, indicating that the predicted RdRp should be a functional molecule and further confirming the presence of the Volzhskoe tick virus in the metagenome.

Bunyaviruses have a single ORF in their M segment that encodes a polyprotein. Co- and post-translational processes transform this polyprotein into functional glycoproteins, Gn and Gc, which play a crucial role in recognizing host receptors^27^. On the M segment, we found a single long ORF, which was as expected. Using this ORF, we predicted the amino acid sequence of the translated protein, which showed the organization of the bunyavirus GPC. The number of transmembrane domains in bunyavirus GPCs is variable. In the Volzhskoe tick virus GPC, we found three transmembrane helices: two are located around the middle of the polyprotein, namely at the C-terminus of Gn. The third domain is located at the Gc's C-terminus. The NSm protein was not presented in the sequence of the GPC, although not all bunyaviruses encode non-structural proteins in their M genomic segment^16^. We also detected a signal protein in the N-terminal region of the GPC, a common feature for members of the class *Bunyaviricetes*. Proprotein cleavage sites were predicted at positions 263 and 683, which may play a role in polyprotein processing as several enzymes, including signalases, proteases, furin, subtilisin/kexin isozyme-1/site-1 protease (SKI-1/S1P), and convertases, are involved in the proteolytic processing of bunyavirus GPC^28^. These results support the hypothesis that this previously uncharacterized virus is capable of infecting and replicating in tick cells based on the sequenced L and M genomic segments. The characteristics of the persistence of this virus in nature, potentially without having all three genome segments, remains unclear. However, the publication of high-quality genomic data paves the way for future studies utilizing minigenome systems to explore gene functions and specific aspects of the virus life cycle.

The Volzhskoe tick virus showed a close phylogenetic relationship with other unclassified species of the *Bunyaviricetes*; the most closely related species were also found in various tick species. These included *Ixodes ricinus* with European, *Ixodes scapularis* with North-American and *Hy. marginatum* with palearctic geographical distribution. The importance of providing sequencing data for these ticks is becoming more relevant in light of disease emergence, which is strongly correlated with the population dynamics of the vector, and one of the main benefits of metagenomic surveillance is that we are able to detect coinfections, leading to a deeper understanding of viral interactions.

In addition to the importance of our metagenomic study and the discovery of tick-specific viruses, it also has some limitations. First, the metagenomic approach did not allow us to further classify the Volzhskoe tick virus because its closest relatives were also unclassified. In the future, we would need to isolate and sequence this virus to determine if it has an S segment. Moreover, we are not able to determine based on the sequence information alone whether the Volzhskoe tick virus can infect vertebrates or if it is a tick-specific virus. Based on the current data, we suspect that the Volzhskoe tick virus is most likely a tick-specific virus (ISV), although very little information is available. Obtaining more genomic and ecological data on the virus and its closest relatives is necessary to understanding its potential pathogenicity to humans or animals. Traditional in vitro experiments could also be useful in tick cocultures as well as the serological screening of known hosts of *Hyalomma marginatum*.

In conclusion, our high-throughput metagenomic analyses provide the first genomic and phylogenetic data about the presence of Volzhskoe tick virus in Hungary, a recently reported tick-borne *Bunyaviricetes* member with unknown pathogenicity and host range. Documentation of this virus was possible with the help of volunteer citizen scientists who provided *Hyalomma* spp. specimens for molecular and metagenomic analysis. To take preventive measures against ticks and tick-borne pathogens applying the DAMA (Document, Assess, Monitor, Act) protocol we will continue with the tick monitoring project in the future as it has demonstrated the ability of citizen science to serve pathogen discovery and monitoring and the anticipation of emerging infectious diseases.

## Materials and methods

### Sample collection

As part of a Citizen Science project (https://www.kullancsfigyelo.hu), we collected ticks from April to December 2021. Ticks were reported via email images or by delivering them personally or through mail to the Institute of Evolution, HUN-REN Centre for Ecological Research. The website of the project offered guidance on the proper removal, storage, and mailing of tick specimens^20^. Two specimens stood out due to their larger size and leg stripes; one was identified as *Hyalomma marginatum*. The tick was discovered in a garden of the small town Bük (47° 23′ 09.2″ N 16° 44′ 29.8″ E) in Western Hungary (Vas County) on August 10 2021. The tick was crawling on a dog’s leg when the submitter noticed it. The tick was kept at 4 °C and examined alive, then stored at − 80 °C and subjected to viral metagenomic sequencing.

### Sample processing and sequencing

We subjected the whole tick to bulk RNA sequencing using a previously described protocol^27^. Briefly, the tick was placed into a 2 ml Eppendorf tube with six pieces of steel beads. Two freeze–thaw cycles were applied using liquid nitrogen. The whole individual was crushed with a TissueLyser (Qiagen, Hilden, Germany). We added 500 µl of DMEM to homogenize the sample and then filtered it through a 0.45 µm pore-size column. Viral enrichment was conducted with a mixture of nuclease treatments (Turbo DNase, Ambion, Carlsbad, CA, USA; Baseline-ZERO, Epicenter, Madison, WI, USA; Benzonase, Novagen, San Diego, CA, USA; RNAse One, Promega, Fitchburg, WI, USA). The RNA was extracted with QiampViral RNA (Qiagen, Hilden, Germany). After the sequence-independent RT-PCR amplification, the sequencing library was prepared with the QIAseq FX DNA Library Kit (Qiagen, Hilden, Germany). Normalized samples were pooled and sequenced using 300-cycle (2 × 150 bp paired-end) NextSeq 2000 reagent kits v2.5 (Illumina, San Diego, CA, USA) on a NextSeq 2000 platform. After quality and length filtering, 7,546,102 reads were subjected to de novo assembly in CLC Genomics Workbench. For the metagenomic classification, the DIAMOND + MEGAN pipeline^29^ was applied, using the non-redundant protein database (NCBI, 23.09.2021) [^30,31^]. The curated sequence data were compared with viral databases and nonredundant proteins (https://www.ncbi.nlm.nih.gov/refseq/about/nonredundantproteins; https://www.ncbi.nlm.nih.gov/genome/viruses/). The cut-off E-value for the BLASTx analyses and comparison was set to 0.001. The viral metatranscriptomic output was visualized and analyzed in MEGAN. The generated data are available in GenBank (OQ849227, OQ849228) and the SRA database under accession number SRR24216213.

### Sequence analysis

Geneious v9.1.8 (Biomatters, Auckland, New Zealand) was used to perform reference-guided de novo genome assembly, sequence analysis, and genomic organization. The accession number of references used to separate the Volzhskoe tick virus reads were MN542370, MN542371 and BWA^32^ for the mapping step. Predictions of the open reading frames (ORFs) and amino acid sequences were performed using the Geneious and Expasy translate tool^33^. We used MUSCLE^34^ for multiple alignments, MEGA 11^35^, and Geneious for visualization of our sequence alignments. Protein domain organization and targeting predictions were generated using InterProScan^36^ database, PredictProtein^37^, NetOGlyc 4.0^38^, SignalP 5.0^39^, ProP 1.0^40^, and for iterative searches we used JackHMMER^41^. Transmembrane domain predictions were performed using DeepTMHMM^42^.

### Phylogenetic analysis

Protein sequences of representatives of the class *Bunyaviricetes* were downloaded (January 2023) from GenBank (Table S1, Table S2) and aligned with the predicted amino acid sequences of the Volzhskoe tick virus using MEGA 11 and MUSCLE. Phylogenetic trees were predicted by MEGA 11 with the maximum likelihood method using WAG + Γ + F as the best model (Figure S2). The smaller phylogenetic trees were generated for both genome segments using closely related sequences from the same branch and tested with 1000 bootstrap replicates. The M segments of the species LG + Γ and the L segments LG + Γ + F were used as the model. The visualization of the resulting trees was performed using the online tool iTOL^43^. Pairwise identity plots were generated with an in-house R script using the bio3d and tidyverse packages.

### Supplementary Information

Supplementary Information.

## Data Availability

Data is provided within the manuscript or supplementary information files.
