# Supplementary material for: Genomic characterization of Volzhskoe tick virus (Bunyaviricetes) from a Hyalomma marginatum tick, Hungary
Source: Sci Rep. 2024 Aug 15;14:18945. doi: 10.1038/s41598-024-69776-8 (PMC11327328; doi:10.1038/s41598-024-69776-8)
Supplement: Supplementary file 1 — Supplementary Information. [file 41598_2024_69776_MOESM1_ESM.docx]

| Accession | Species | Type | Gene | Family | Country | Host | Link |
| --- | --- | --- | --- | --- | --- | --- | --- |
| QPD01627 | *Volzhskoe tick virus* | prot | GPC | Unclass | Russia | *Hyalomma marginatum* | https://www.ncbi.nlm.nih.gov/nuccore/QPD01627 |
| USL85430 | *Ixodes ricinus bunyavirus-like virus 1* | prot | GPC | Unclass | Croatia | Ixodes ricinus | https://www.ncbi.nlm.nih.gov/nuccore/USL85430 |
| USL85420 | *Bronnoya virus* | prot | GPC | Unclass | Croatia | Ixodes ricinus | https://www.ncbi.nlm.nih.gov/nuccore/USL85420 |
| ASY03251 | *Bronnoya virus* | prot | GPC | Unclass | Norway | Ixodes ricinus | https://www.ncbi.nlm.nih.gov/nuccore/ASY03251 |
| BBD75426 | *Ixodes scapularis bunyavirus* | prot | GPC | *Peribunyaviridae* | Japan | na | https://www.ncbi.nlm.nih.gov/nuccore/BBD75426 |
| YP010084299 | *California encephalitis orthobunyavirus* | prot | GPC | *Peribunyaviridae* | na | na | https://www.ncbi.nlm.nih.gov/nuccore/YP010084299 |
| YP010085089 | *Leptomonas shilevirus* | prot | GPC | *Leishbuviridae* | na | na | https://www.ncbi.nlm.nih.gov/nuccore/YP010085089 |
| YP010086061 | *Salehabad phlebovirus* | prot | GPC | *Phenuiviridae* | na | na | https://www.ncbi.nlm.nih.gov/nuccore/YP010086061 |
| YP009664554 | *Ferak feravirus* | prot | GPC | *Phasmaviridae* | na | na | https://www.ncbi.nlm.nih.gov/nuccore/YP009664554 |
| YP009664558 | *Turlock orthobunyavirus* | prot | GPC | *Peribunyaviridae* | na | na | https://www.ncbi.nlm.nih.gov/nuccore/YP009664558 |
| YP009666905 | *Batai orthobunyavirus* | prot | GPC | *Peribunyaviridae* | na | na | https://www.ncbi.nlm.nih.gov/nuccore/YP009666905 |
| YP009666318 | *Millipede wumivirus* | prot | GPC | *Wupedeviridae* | na | na | https://www.ncbi.nlm.nih.gov/nuccore/YP009666318 |
| YP009666938 | *Guertu bandavirus* | prot | GPC | *Phenuiviridae* | na | na | https://www.ncbi.nlm.nih.gov/nuccore/YP009666938 |
| YP009667022 | *Witwatersrand orthobunyavirus* | prot | GPC | *Peribunyaviridae* | na | na | https://www.ncbi.nlm.nih.gov/nuccore/YP009667022 |
| YP009667046 | *Tacaiuma orthobunyavirus* | prot | GPC | *Peribunyaviridae* | na | na | https://www.ncbi.nlm.nih.gov/nuccore/YP009667046 |
| YP009507851 | *Hazara orthonairovirus* | prot | GPC | *Nairoviridae* | na | na | https://www.ncbi.nlm.nih.gov/nuccore/YP009507851 |
| YP009512940 | *Punta Toro phlebovirus* | prot | GPC | *Phenuiviridae* | na | na | https://www.ncbi.nlm.nih.gov/nuccore/YP009512940 |
| YP009361837 | *Nairobi sheep disease orthonairovirus* | prot | GPC | *Nairoviridae* | na | na | https://www.ncbi.nlm.nih.gov/nuccore/YP009361837 |
| YP009345126 | *Myriapod hubavirus* | prot | GPC | *Mypoviridae* | na | na | https://www.ncbi.nlm.nih.gov/nuccore/YP009345126 |
| YP009329880 | *Lincruvirus wenlingense* | prot | GPC | *Cruliviridae* | na | na | https://www.ncbi.nlm.nih.gov/nuccore/YP009329880 |
| YP009047241 | *Heartland bandavirus* | prot | GPC | *Phenuiviridae* | na | na | https://www.ncbi.nlm.nih.gov/nuccore/YP009047241 |
| YP003848705 | *Rift Valley fever phlebovirus* | prot | GPC | *Phenuiviridae* | na | na | https://www.ncbi.nlm.nih.gov/nuccore/YP003848705 |
| YP003104765 | *European mountain ash ringspot-associated virus* | prot | GPC | *Fimoviridae* | na | na | https://www.ncbi.nlm.nih.gov/nuccore/YP003104765 |
| YP001497160 | *Akabane orthobunyavirus* | prot | GPC | *Peribunyaviridae* | na | na | https://www.ncbi.nlm.nih.gov/nuccore/YP001497160 |
| YP006504094 | *Dabie bandavirus* | prot | GPC | *Phenuiviridae* | na | na | https://www.ncbi.nlm.nih.gov/nuccore/YP006504094 |
| YP009225 | *Tomato spotted wilt orthotospovirus* | prot | GPC | *Tospoviridae* | na | na | https://www.ncbi.nlm.nih.gov/nuccore/YP009225 |
| NP955542 | *Dugbe orthonairovirus* | prot | GPC | *Nairoviridae* | na | na | https://www.ncbi.nlm.nih.gov/nuccore/NP955542 |
| NP950235 | *rimean-Congo hemorrhagic fever orthonairovirus* | prot | GPC | *Nairoviridae* | na | na | https://www.ncbi.nlm.nih.gov/nuccore/NP950235 |
| NP942586 | *Tula orthohantavirus* | prot | GPC | *Hantaviridae* | na | na | https://www.ncbi.nlm.nih.gov/nuccore/NP942586 |
| NP942554 | *Dobrava-Belgrade orthohantavirus* | prot | GPC | *Hantaviridae* | na | na | https://www.ncbi.nlm.nih.gov/nuccore/NP942554 |
| NP942557 | *Seoul orthohantavirus* | prot | GPC | *Hantaviridae* | na | na | https://www.ncbi.nlm.nih.gov/nuccore/NP942557 |
| NP941983 | *Puumala orthohantavirus* | prot | GPC | *Hantaviridae* | na | na | https://www.ncbi.nlm.nih.gov/nuccore/NP941983 |
| NP941987 | *Hantaan orthohantavirus* | prot | GPC | *Hantaviridae* | na | na | https://www.ncbi.nlm.nih.gov/nuccore/NP941987 |
| NP604472 | *Andes orthohantavirus* | prot | GPC | *Hantaviridae* | na | na | https://www.ncbi.nlm.nih.gov/nuccore/NP604472 |
| NP899218 | *Argentinian mammarenavirus* | prot | GPC | *Arenaviridae* | na | na | https://www.ncbi.nlm.nih.gov/nuccore/NP899218 |
| NP671969 | *La Crosse orthobunyavirus* | prot | GPC | *Peribunyaviridae* | na | na | <https://www.ncbi.nlm.nih.gov/nuccore/NP671969> |
| NP694851 | *Lymphocytic choriomeningitis mammarenavirus* | prot | GPC | *Arenaviridae* | na | na | <https://www.ncbi.nlm.nih.gov/nuccore/NP694851> |

**Table S1** Downloaded representative sequences of GPC of different bunyaviruses.

| Accession | Species | Type | Gene | Family | Country | Host | Link |
| --- | --- | --- | --- | --- | --- | --- | --- |
| BBD75425 | *Ixodes scapularis bunyavirus* | prot | RdRp | *Peribunyaviridae* | Japan | *Ixodes scapularis* | https://www.ncbi.nlm.nih.gov/nuccore/BBD75425 |
| QPB70159 | *Almazovo tick virus* | prot | RdRp | Unclass | Russia | *Ixodes ricinus* | https://www.ncbi.nlm.nih.gov/nuccore/QPB70159 |
| USL85421 | *Bronnoya virus* | prot | RdRp | Unclass | Croatia | *Ixodes ricinus* | https://www.ncbi.nlm.nih.gov/nuccore/USL85421 |
| ASY03250 | *Bronnoya virus* | prot | RdRp | Unclass | Norway | *Ixodes ricinus* | https://www.ncbi.nlm.nih.gov/nuccore/ASY03250 |
| USL85429 | *Ixodes ricinus bunyavirus-like virus 1* | prot | RdRp | Unclass | Croatia | *Ixodes ricinus* | https://www.ncbi.nlm.nih.gov/nuccore/USL85429 |
| QKK82912 | *Ubmeje virus* | prot | RdRp | Unclass | Sweden | *Ixodes uriae* | https://www.ncbi.nlm.nih.gov/nuccore/QKK82912 |
| QPD01626 | *Volzhskoe tick virus* | prot | RdRp | Unclass | Russia | *Hyalomma marginatum* | https://www.ncbi.nlm.nih.gov/nuccore/QPD01626 |
| YP010084300 | *California encephalitis orthobunyavirus* | prot | RdRp | *Peribunyaviridae* | USA | na | https://www.ncbi.nlm.nih.gov/nuccore/YP010084300 |
| YP010085090 | *Leptomonas shilevirus* | prot | RdRp | *Leishbuviridae* | Madagascar | na | https://www.ncbi.nlm.nih.gov/nuccore/YP010085090 |
| YP010086058 | *Salehabad phlebovirus* | prot | RdRp | *Phenuiviridae* | Iran | na | https://www.ncbi.nlm.nih.gov/nuccore/YP010086058 |
| YP009664550 | *Ferak feravirus* | prot | RdRp | *Phasmaviridae* | Cote d Ivoire | na | https://www.ncbi.nlm.nih.gov/nuccore/YP009664550 |
| YP009664559 | *Turlock orthobunyavirus* | prot | RdRp | *Peribunyaviridae* | India | na | https://www.ncbi.nlm.nih.gov/nuccore/YP009664559 |
| YP009666319 | *Millipede wumivirus* | prot | RdRp | *Wupedeviridae* | China | na | https://www.ncbi.nlm.nih.gov/nuccore/YP009666319 |
| YP009666906 | *Batai orthobunyavirus* | prot | RdRp | *Peribunyaviridae* | Malaysia | na | https://www.ncbi.nlm.nih.gov/nuccore/YP009666906 |
| YP009666941 | *Guertu bandavirus* | prot | RdRp | *Phenuiviridae* | China | na | https://www.ncbi.nlm.nih.gov/nuccore/YP009666941 |
| YP009667021 | *Witwatersrand orthobunyavirus* | prot | RdRp | *Peribunyaviridae* | South Africa | na | https://www.ncbi.nlm.nih.gov/nuccore/YP009667021 |
| YP009667044 | *Tacaiuma orthobunyavirus* | prot | RdRp | *Peribunyaviridae* | Brazil | na | https://www.ncbi.nlm.nih.gov/nuccore/YP009667044 |
| YP009507850 | *Hazara orthonairovirus* | prot | RdRp | *Nairoviridae* | Pakistan | na | https://www.ncbi.nlm.nih.gov/nuccore/YP009507850 |
| YP009361832 | *Nairobi sheep disease orthonairovirus* | prot | RdRp | *Nairoviridae* | China | na | https://www.ncbi.nlm.nih.gov/nuccore/YP009361832 |
| YP009512941 | *Punta Toro phlebovirus* | prot | RdRp | *Phenuiviridae* | Panama | na | https://www.ncbi.nlm.nih.gov/nuccore/YP009512941 |
| YP009345128 | *Myriapod hubavirus* | prot | RdRp | *Mypoviridae* | China | na | https://www.ncbi.nlm.nih.gov/nuccore/YP009345128 |
| YP009329879 | *Lincruvirus wenlingense* | prot | RdRp | *Cruliviridae* | China | na | https://www.ncbi.nlm.nih.gov/nuccore/YP009329879 |
| YP009047242 | *Heartland bandavirus* | prot | RdRp | *Phenuiviridae* | USA | na | https://www.ncbi.nlm.nih.gov/nuccore/YP009047242 |
| YP006504091 | *SFTS phlebovirus* | prot | RdRp | *Phenuiviridae* | China | na | https://www.ncbi.nlm.nih.gov/nuccore/YP006504091 |
| YP003848704 | *Rift Valley fever phlebovirus* | prot | RdRp | *Phenuiviridae* | Egypt | na | https://www.ncbi.nlm.nih.gov/nuccore/YP003848704 |
| YP003104764 | *European mountain ash ringspot-associated emaravirus* | prot | RdRp | *Fimoviridae* | na | na | https://www.ncbi.nlm.nih.gov/nuccore/YP003104764 |
| YP001497159 | *Akabane orthobunyavirus* | prot | RdRp | *Peribunyaviridae* | na | na | https://www.ncbi.nlm.nih.gov/nuccore/YP001497159 |
| YP325663 | *Crimean-Congo hemorrhagic fever orthonairovirus* | prot | RdRp | *Nairoviridae* | na | na | https://www.ncbi.nlm.nih.gov/nuccore/YP325663 |
| NP942555 | *Dobrava-Belgrade orthohantavirus* | prot | RdRp | *Hantaviridae* | Greece | na | https://www.ncbi.nlm.nih.gov/nuccore/NP942555 |
| NP942558 | *Seoul orthohantavirus* | prot | RdRp | *Hantaviridae* | na | na | https://www.ncbi.nlm.nih.gov/nuccore/NP942558 |
| NP942124 | *Tula orthohantavirus* | prot | RdRp | *Hantaviridae* | na | na | https://www.ncbi.nlm.nih.gov/nuccore/NP942124 |
| NP941976 | *Sin Nombre orthohantavirus* | prot | RdRp | *Hantaviridae* | na | na | https://www.ncbi.nlm.nih.gov/nuccore/NP941976 |
| NP941982 | *Hantaan orthohantavirus* | prot | RdRp | *Hantaviridae* | na | na | https://www.ncbi.nlm.nih.gov/nuccore/NP941982 |
| NP899217 | *Argentinian mammarenavirus* | prot | RdRp | *Arenaviridae* | na | na | https://www.ncbi.nlm.nih.gov/nuccore/NP899217 |
| NP671968 | *La Crosse orthobunyavirus* | prot | RdRp | *Peribunyaviridae* | na | na | https://www.ncbi.nlm.nih.gov/nuccore/NP671968 |
| NP604473 | *Andes orthohantavirus* | prot | RdRp | *Hantaviridae* | na | na | https://www.ncbi.nlm.nih.gov/nuccore/NP604473 |
| NP694851 | *Lymphocytic choriomeningitis mammarenavirus* | prot | RdRp | *Arenaviridae* | Arenaviridae | na | https://www.ncbi.nlm.nih.gov/nuccore/NP694851 |
| NP690576 | *Dugbe orthonairovirus* | prot | RdRp | *Nairoviridae* | na | na | https://www.ncbi.nlm.nih.gov/nuccore/NP690576 |
| NP694845 | *Lymphocytic choriomeningitis mammarenavirus* | prot | RdRp | *Arenaviridae* | na | na | https://www.ncbi.nlm.nih.gov/nuccore/NP694845 |
| NP049362 | *Tomato spotted wilt orthotospovirus* | prot | RdRp | *Tospoviridae* | na | na | https://www.ncbi.nlm.nih.gov/nuccore/NP049362 |
| QDF45211 | *Sandfly fever Naples phlebovirus* | prot | RdRp | *Phenuiviridae* | Switzerland | na | https://www.ncbi.nlm.nih.gov/nuccore/QDF45211 |
| AWH61702 | *Uukuniemi phlebovirus* | prot | RdRp | *Phenuiviridae* | Czech Republic | na | https://www.ncbi.nlm.nih.gov/nuccore/AWH61702 |
| AFH89032 | *Erve orthonairovirus* | prot | RdRp | *Nairoviridae* | na | na | https://www.ncbi.nlm.nih.gov/nuccore/AFH89032 |

**Table S2** Downloaded representative sequences of RdRp of different bunyaviruses.

| Accession | Species | Family | Gene | Link |
| --- | --- | --- | --- | --- |
| ASY03250 | *Bronnoya virus* | Unclass | RdRp | https://www.ncbi.nlm.nih.gov/nuccore/ASY03250 |
| QKK82912 | *Ubmeje virus* | Unclass | RdRp | https://www.ncbi.nlm.nih.gov/nuccore/QKK82912 |
| QPB70159 | *Almazovo tick virus* | Unclass | RdRp | https://www.ncbi.nlm.nih.gov/nuccore/QPB70159 |
| QPD01626 | *Volzhskoe tick virus* | Unclass | RdRp | https://www.ncbi.nlm.nih.gov/nuccore/QPD01626 |
| QSW03630 | *Lichuan virus* | Unclass | RdRp | https://www.ncbi.nlm.nih.gov/nuccore/QSW03630 |
| P20470 | *Bunyawera virus* | Peribunyaviridae | RdRp | https://www.ncbi.nlm.nih.gov/nuccore/P20470 |
| P23456 | *Hantaan virus* | Hantaviridae | RdRp | https://www.ncbi.nlm.nih.gov/nuccore/P23456 |
| P28976 | *Tomato spotted wilt virus* | Tospoviridae | RdRp | https://www.ncbi.nlm.nih.gov/nuccore/P28976 |
| P33453 | *Uukuniemi virus* S23 | Phenuiviridae | RdRp | https://www.ncbi.nlm.nih.gov/nuccore/P33453 |
| Q85431 | *Rice stripe virus* | Phenuiviridae | RdRp | https://www.ncbi.nlm.nih.gov/nuccore/Q85431 |
| Q8JPR2 | *La Crosse virus* L78 | Peribunyaviridae | RdRp | https://www.ncbi.nlm.nih.gov/nuccore/Q8JPR2 |

**Table S3** Downloaded RdRp sequences of different bunyaviruses used for the motif searching


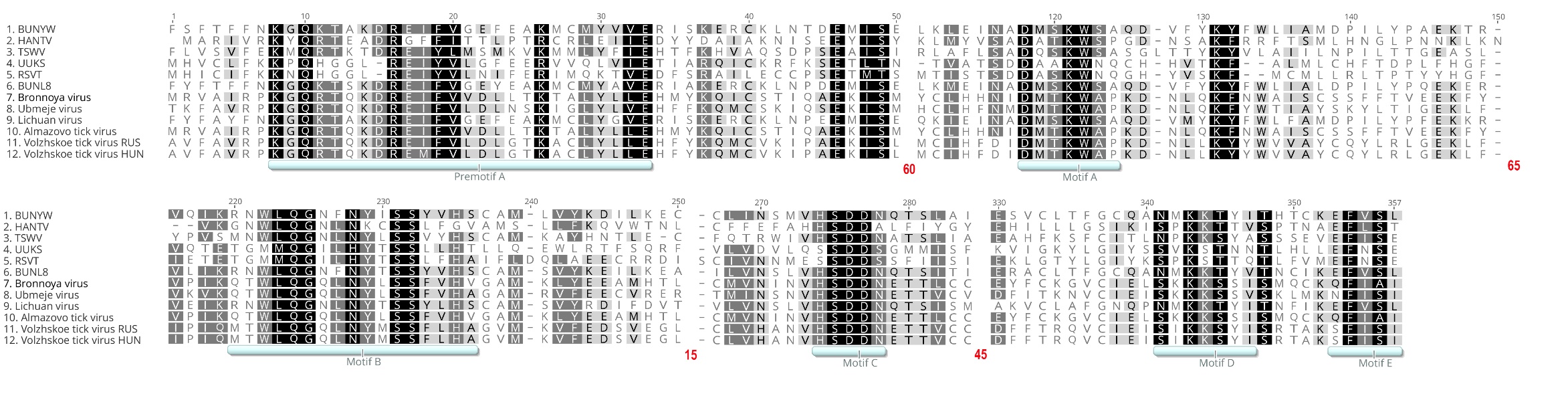


**Figure S1** The RdRp of Volzhskoe tick virus and other bunyaviruses display conserved motifs A to E and premotif A. The selected bunyaviruses: BUNYW – Bunyawera virus(P20470), HANTV – Hantaan virus (P23456), TSWV – Tomato spotted wilt virus (P28976), UUKS – Uukuniemi virus S23 (P33453), RSVT – Rice stripe virus (Q85431) and BUNL8 – La Crosse virus L78 (Q8JPR2). The most closely related species of unclassified bunyaviruses are indicated by numbers 7-11. The red numbers below the alignment indicate the number of deleted positions.


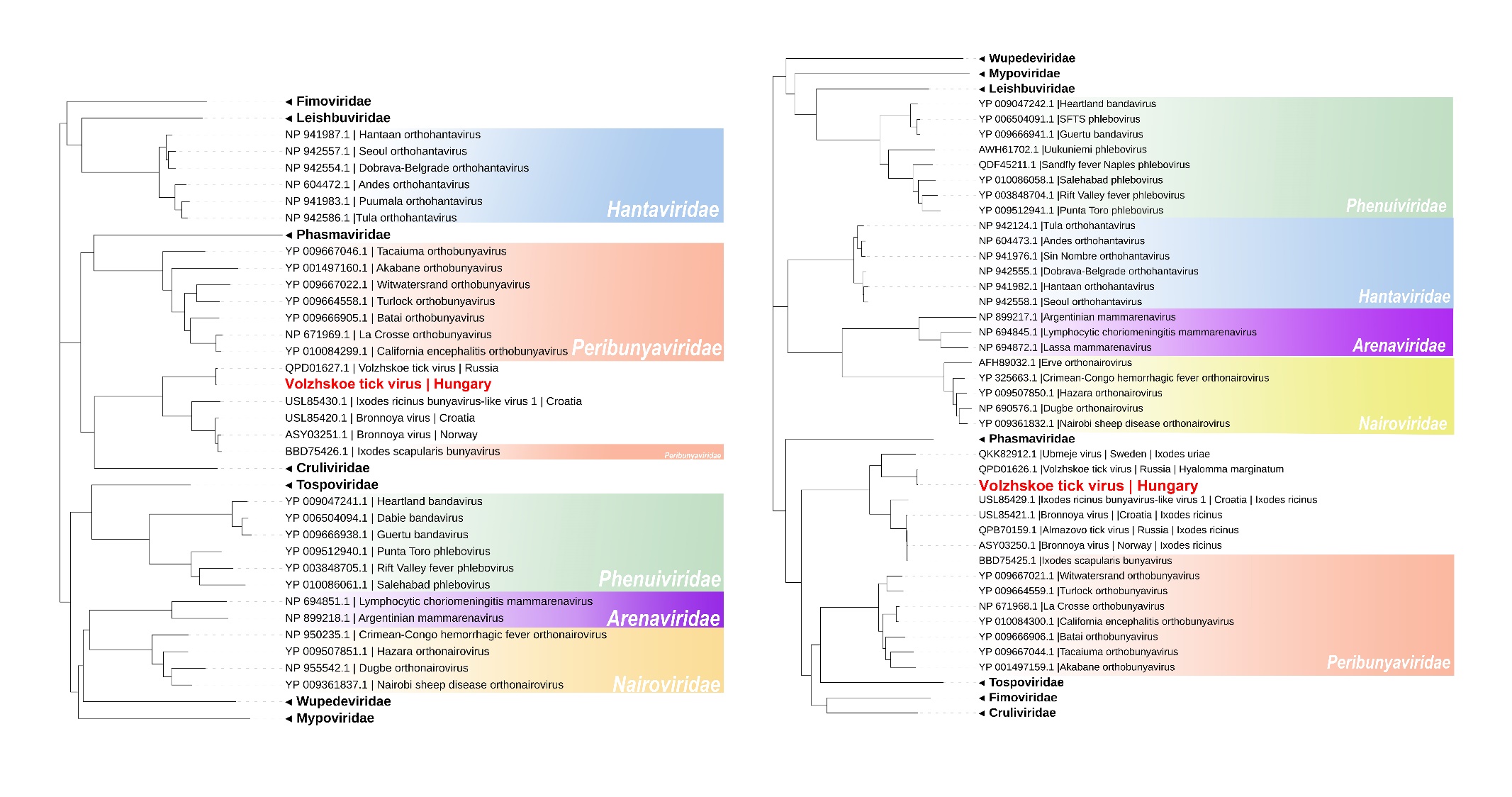


**Figure S2** Maximum likelihood phylogenetic trees based on the segment M (A) and segment L (B) of representative *Bunyaviricetes* and the newly identified Volzhskoe tick virus (red). The trees were coloured by families.
